# Cautious Sexual Attitudes Diminish Intent to Vaccinate Children against HPV in Utah

**DOI:** 10.3390/vaccines10091382

**Published:** 2022-08-24

**Authors:** David S. Redd, Jamie L. Jensen, Ruth J. Bodily, Abigail A. Lee, Ty J. Skyles, Brian D. Poole

**Affiliations:** 1 Department of Microbiology and Molecular Biology, Brigham Young University, Provo, UT 84602, USA; 2 Department of Biology, Brigham Young University, Provo, UT 84602, USA

**Keywords:** vaccine hesitancy, human papillomavirus, religion and society

## Abstract

Although most Human papillomavirus (HPV) infections are mild and are cleared by the immune system, some high-risk HPV strains can cause various cancers. Vaccines have been developed that protect against high-risk HPV strains. HPV vaccines have been approved for use by the CDC (Centers for Disease Control and Prevention) and are recommended for everyone aged 11–26. Despite the availability of safe and effective vaccines, uptake is low. HPV vaccine uptake has been extensively studied on a national and international level, but less is known about vaccine acceptance on a state or local level. The state of Utah, in the United States of America, has a relatively low HPV vaccination rate. In this study, we identified factors that impact the intent of Utah parents to vaccinate their children against HPV. A survey was distributed electronically to Utah residents. Survey results were analyzed using confirmatory factor analysis, structural equation modeling, and univariate analysis. Knowledge about HPV and positive vaccine attitudes had the greatest positive effect on intent to vaccinate children against HPV. Cautious sexual attitudes and high religious practice were found to have a negative impact on intent to vaccinate. Effective public health messaging will consider the cultural and religious influences of the target population.

## 1. Introduction

Human papillomaviruses (HPV) are a family of over 150 related human viruses. HPV are non-enveloped double stranded DNA viruses. HPV have co-evolved with humans over millions of years, and are therefore well adapted, often causing minimal symptoms or being completely asymptomatic [1]. HPV infects cutaneous and mucosal stratified epithelial cells. In order to replicate, HPV promotes cell cycle progression within infected cells. The modulation of the cell cycle can cause increased cell proliferation resulting in growths called warts or papillomas [2]. Although most HPV infections are mild and typically clear on their own within a few months to a few years, persistent HPV infections are strongly associated with the development of various cancers [3,4]. HPV causes 99% of cervical cancers, 90% of anal cancers 50–65% of vulvar and vaginal cancers, and between 45% and 90% of oropharyngeal cancers [5]. This virus family is responsible for approximately 5% of the world’s total cancer burden [6].

HPV is the most commonly sexually transmitted infection in the world. Between 50–80% of individuals will be infected with HPV sometime during their life [3,7]. The virus is generally transmitted through skin to skin or sexual contact. Although HPV is generally sexually transmitted evidence suggests that HPV can also be transmitted on surfaces or medical implements [5]. Many common clinical disinfectants are ineffective against HPV and it can remain active on surfaces even after cleaning [8].

Due to the potential harm that HPV can cause, significant effort was made to develop preventative vaccines. Three vaccines have been approved for use in the United States by the Food and Drug administration (FDA). All three vaccines provide protection against the oncogenic HPV strains 16 and 18, which are responsible for 70% of HPV induced cervical cancers [3]. Multiple studies have shown that HPV vaccines are safe for human use, and effective at reducing infection with high-risk HPV strains [6]. There has been measurable reduction in the incidence and transmission of HPV strains covered by vaccines [9,10].

Despite the effectiveness of HPV vaccines at preventing HPV infection and progression into various cancers, vaccine uptake is low. In 2018 global HPV vaccine uptake was estimated to be 12.2% for 15 year-old females. There are several reasons for low uptake, including demographics, financial constraints, and fear of side effects [11,12,13]. Methods to improve vaccine acceptance include provider recommendation [14,15] and culturally appropriate communication [16]. Significant variation in vaccination rates was observed between wealthy and developing nations, with wealthy nations reporting higher vaccination rates [17]. Although HPV vaccination rates in the United States are higher than the global average, vaccine uptake has been modest. A 2016 study reported that 60% of adolescents 13–17 have received at least 1 vaccine dose and that only 37% have completed the vaccine series [18]. Young adults (18–26) have an even lower vaccination rate than adolescents. The US department of health and Human Services (HHS) estimates that half of young adults have received at least 1 vaccine dose and less than 22% have completed a vaccine series [19].

There have been many studies assessing HPV vaccine uptake at a national level but there has been little research done about what factors impact HPV vaccination rates at a state level. For the past 5 years, the percentage of Utah adolescents who have received the recommended number of HPV vaccine doses has been persistently among the lowest in the country (approximately 45% of eligible teens, rank 41 out of 50 states) [17,20]. The studies on HPV vaccination in Utah that have been performed focused primarily on health care providers [21,22]. In one of these studies health care providers considered parental misconceptions to be the strongest barrier to HPV vaccination in Utah [23]. Another Utah study found that religious young women are underinformed about HPV and are under-vaccinated [23]. Utah is an interesting state to look at how cultural and religious factors influence HPV vaccination attitudes. A large portion of the state is highly religious, with the dominant religion strongly emphasizing sexual abstinence before marriage. The population is relatively young, and there is a mix of rural and urban inhabitants. To better understand what factors impact parental intent to vaccinate their children, we surveyed Utah parents directly.

## 2. Materials and Methods

### 2.1. Survey of Utah Residents

**Figure 1:**
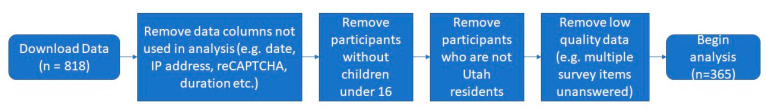
Pre-analysis data cleaning: We downloaded data collected by Qualtrics. Data columns that contained data that would not be used in analysis were removed (e.g., start date, end date, IP address, reCAPTCHA, etc.) Next responses of participants who did not meet our exclusion criteria (Utah resident and have a child 15 or younger) were removed. Low quality data was also removed (e.g., multiple survey items left unanswered, outcome variables left blank) Cleaning reduced our sample size from 818 to 365.

Utah residents were invited to participate in a cross-sectional study by completing a survey designed to assess attitudes toward HPV vaccination. The survey was distributed through an online portal by Qualtrics (Provo, UT, USA). Initial inclusion criteria required participants to be a current resident of Utah. Survey results were later filtered so only parents with a child 15 or under were included in our sample, to assess those who are making HPV vaccination decisions (Figure 1). To minimize bias in online survey participants, who tend to be highly educated, filters were applied to collect responses from educational backgrounds consistent with census data for Utah.

### 2.2. Survey Description

The survey contained 80 questions in total. Some questions were clarifying questions and were only shown if certain responses were chosen on previous questions, such as questions 2.15–2.21, which clarify specific religious denominations, and questions 3.6 and 3.7 clarifying which news sources are most commonly used. The questions were divided into 10 sections. The first section contained questions about demographics. The second section assessed primary sources of news. The third and fourth sections assessed trust in government and modern medicine, respectively. In the fifth section participants were asked about their religious practice. The sixth section assessed aspirations for their children. In the seventh section, participants were asked about their sexual attitudes and what they intended to teach their children about sex. The eighth section assessed knowledge about HPV. The ninth section assessed participants’ attitudes towards vaccines in general. The final section contained outcome questions which tested the attitudes of parents toward HPV and their intent to vaccinate their children against it. Prior to distribution the study received ethical approval from the institutional review board of Brigham Young University (IRB# IRB2022-165). The entire survey is available in the Supplementary Material (Table S1).

### 2.3. Confirmatory Factor Analysis and Structural Equation Modeling

To validate our survey, confirmatory factor analysis (CFA) was used to confirm the latent variables we were testing were accurately represented by the questions we chose to include in the survey. We preformed structural equation modeling (SEM) to test the relationship between variables. Before starting analyses we cleaned and organized the data using Excel (Microsoft 2022 Redmond, Redmond, WA, USA) and SPSS statistics software (IBM 2021 Armonk, NY, USA). The first step of cleaning the data was removing participants who indicated that they did not have children, because we wanted to look at what factors impacted parental intent to vaccinate their children. Next we removed data determined to be incomplete or low quality. Mplus software, ver 8 (Muthen and Muthen, 1998–2001, Los Angeles, CA, USA) was used to perform CFA and SEM on the measurement and structural portions of our model. Latent variables were represented by three or more survey items. CFA was performed with a request for modification indices. Survey items representing latent variables were removed until fit indices (root mean square error approximation (RMSE), comparative fit index (CFI), Tucker–Lewis index (TLI) and standardized root mean square residual (SRMR)) were found to be acceptable. SEM was performed on a complete model comprised of all the validated latent variables with age, gender, income and education as covariates. The validated latent variables were divided to form two hypothetical models, the first to test the relationship between trust in medical professionals and religious practices on intent to vaccinate through general vaccine attitudes; and the second to test the relationship between knowledge of HPV and religious practice on intent to vaccinate through sexual attitudes. SEM was performed on the hypothetical models.

### 2.4. Univariate Analysis

Univariate analysis and data visualization were performed to further explore the factors that impact intent to vaccinate that were identified by structural equation modeling. To illustrate our sample population’s attitude toward HPV vaccination we created stacked bar charts of our outcome variables using the *likert*, *tidyverse* and *ggplot2* libraries in R. We used the *corrplot* and *psych* R libraries to create a series of correlation matrixes to determine which variables were correlated with intent to vaccinate.

## 3. Results

### 3.1. Demographic Characteristics of Survey Respondents

We summarized demographic characteristics of our sample (Table 1) before beginning formal analysis of our dataset. We selected our dataset to only include respondents with children. Most respondents had 1 or 2 children (68.5%). The vast majority of respondents were between 26 and 45 years old (82%). Approximately one third of our sample identified as male (31.1%) and two thirds identified as female (68.9%). Participants had the ability to select “Non-binary/third gender” or “I prefer not to answer” for Gender. One participant declined to answer and no participants selected Non-binary/third gender so these choices were removed from the table for clarity. The vast majority of participants selected white (83%) as their ethnicity. More than half of the participants indicated that they were married (68.4%). Almost all of the participants in our study have completed high school (97.5%) but less than half have completed a college degree (44.65%). A vast majority of our participants have a yearly household income less than $100,000 (82.4%). In our sample 37.6% of participants identified as Republican, 33.5% identified as independent, and 19.0% identified as Democrat. Political leanings on social issues had a relatively even distribution, with the largest portion (36.7%) of respondents indicating that they are neither liberal nor conservative leaning. A majority of respondents in our sample indicated they were Christian (57.81%). The second largest group indicated that they had no religious affiliation (27.67%).

### 3.2. Confirmatory Factor Analysis

**Figure 2:**
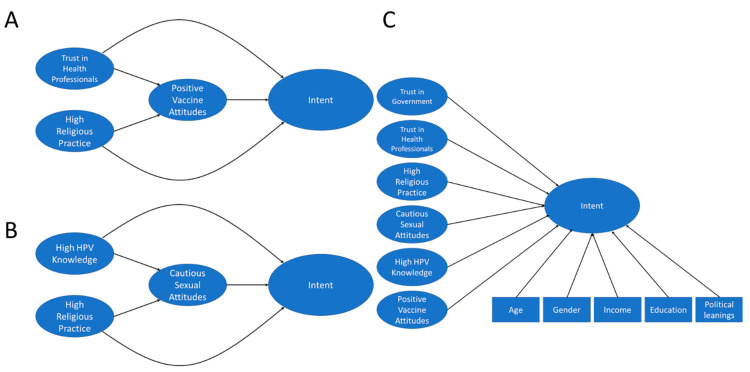
Hypothetical structural models: (**A**) We hypothesize that trust in health professionals and high religious practice negatively influence positive vaccine attitudes which in turn influences intent to vaccinate. We also hypothesize that trust in health professionals and high religious practice may influence intent to vaccinate directly. (**B**) We hypothesize that high HPV knowledge and high religious practice influence cautious sexual attitudes which in turn influences intent to vaccinate. We also hypothesize that high HPV knowledge and high religious practice could influence intent to vaccinate directly. (**C**) We hypothesize that the latent variables trust in government, trust in health professionals, high religious practice, cautious sexual attitudes, high HPV knowledge, and positive vaccine attitudes may directly influence intent to vaccinate. We also hypothesize that the covariates age, gender, income, education and political leanings may impact intent to vaccinate.

CFA models were run for the latent variables included in the hypothetical structural models used for SEM. Items 4.1.1 (“I always vote in national elections”) and 4.1.2 (“I always vote in local elections”) were removed from the latent variable “trust in government” because they did not fit with the other variables using the confirmatory factor analysis concerning trust in government. Two items were removed from the latent variable “trust in health professionals” due to lack of fit (5.1.3 “Natural remedies such as essential oils are as effective at treating most conditions as prescription drugs” and 5.1.5 “Natural remedies are often a better treatment for minor ailments than modern medicine”). No items were removed from “religious practice.” The questions included in “aspirations for children” did not form a single latent variable and therefore were not included in CFA or SEM. Items 8.1 (“As a parent, I emphasize certain rules or cautions about sexual behavior”) and 8.6 (“Sexually transmitted infections are very concerning to me”) did not fit with the latent variable “cautious sexual attitudes” and were therefore removed. Item 9.1.3 “HPV causes cancer in women but not men” was removed from the latent variable “high HPV knowledge” due to lack of fit. Survey item 9.2 (“Vaccines often have severe side effects”) was removed from the latent variable “Positive vaccine attitudes” due to lack of fit. All the items used to determine “intent to vaccinate” fit the model with acceptable fit statistics (Table 2) (Figure 2).

### 3.3. Structural Equation Modeling

**Figure 3:**
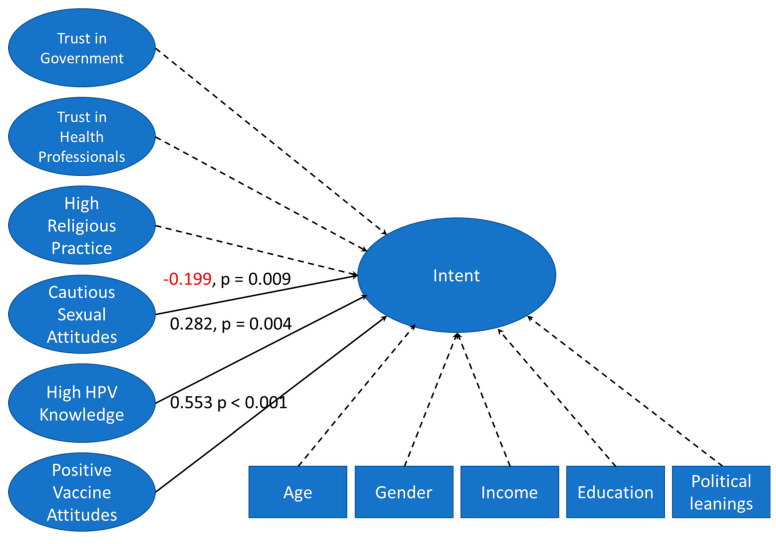
Full structural model: Latent variables and covariates were combined into a full structural model. Structural equation modeling was performed to determine which factors impact parental intent to vaccinate their children against HPV. More cautious sexual attitudes lead to lower intent. Higher knowledge about HPV leads to higher intent to vaccinate. The more positively parents view vaccines the higher their intent to vaccinate their children against HPV. All other latent variables and covariates were not significantly predictive of intent to vaccinate in the full structural model. Values are beta coefficients. Value in red shows negative correlation.

All the latent variables verified by CFA were combined into a single model with the covariates income, education, age, gender, and political leanings on social issues (Figure 3). SEM on the combined model shows a robust fit, as indicated by fit statistics and probability scores (Table 3). Structural equation modeling was used to determine which covariates and latent variables influenced intent to vaccinate in the combined model. The combined model indicates that respondents with more cautious sexual attitudes have lower intent to vaccinate (−0.199). The model also indicates that participants with high knowledge about HPV and positive attitudes towards vaccination have a higher intent to vaccinate (0.282, 0.553, respectively) Positive vaccine attitudes had the greatest effect on intent to vaccinate of all the latent variables tested in the combined model. In the combined model there is not a significant relationship between trust in government, trust in health professionals, high religious practice, and intent to vaccinate. The covariates age, gender, income, education, and political leanings on social issues did not significantly impact intent to vaccinate in the combined model.

**Figure 4:**
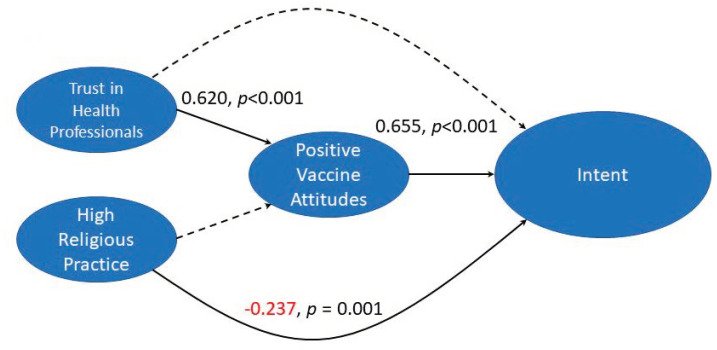
Trust in medical professionals is predictive of intent to vaccinate by means of increased trust in vaccines in general: The Latent variables “Trust in Health Professionals”, “High religious practice”, “Positive vaccine attitudes”, and “Intent to vaccinate” were combined in a structural model. Trust in health professionals leads to more positive vaccine attitudes, which increased intent to vaccinate. High religious practice does not affect general vaccine attitudes, but it does have a direct negative impact on intent to vaccinate against HPV. Values shown are beta coefficients. Value in red shows negative correlation.

SEM on model A shows a robust fit, as indicated by fit statistics (Table 3). The model (Figure 4) indicates that high trust in health professionals leads to more positive vaccine attitudes (+0.620) which increases intent to vaccinate (+0.665). The indirect effect through positive vaccine attitudes explains 96% of the effect of trust in health professionals on intent to vaccinate. High religious practice does not affect general vaccine attitudes in this model, but it does have a negative impact on intent to vaccinate against HPV. This indicates that in our sample, high religious practice impacts general intent to vaccinate and intent to vaccinate against HPV differently.

**Figure 5:**
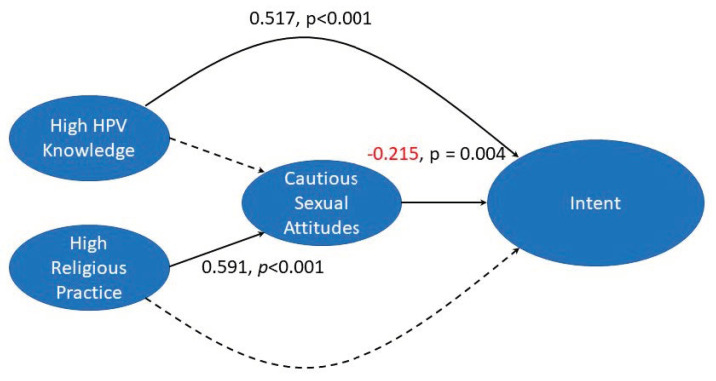
Cautious sexual attitudes negatively impact intent to vaccinate for HPV. The latent variables “High HPV Knowledge”, “High Religious Practice”, “Cautious Sexual Attitudes” and “Intent to vaccinate” were combined in a structural model. Higher HPV knowledge has no significant effect on sexual attitudes but does directly impact intent to vaccinate. Religious practice does not directly impact intent to vaccinate when combined with the other latent variables in this model. Religious practice influences sexual attitudes which negatively influences intent to vaccinate. Values are beta coefficients. Value in red shows negative correlation.

SEM on Model B also shows a robust fit, as indicated by fit statistics (Table 3). The model (Figure 5) indicates that higher knowledge about HPV has no effect on sexual attitudes but does directly impact intent to vaccinate against HPV. In this model the impact of religious practice on intent to vaccinate against HPV, which was shown in the previous model, was found to be mediated through the effect of high religious practice on cautious sexual attitudes (+0.591) which negatively impacts intent to vaccinate (−0.215). In other words, the higher a participant’s religious practice, the more cautious they are about sex, and the more cautious they are, the less likely they are to vaccinate against HPV. The indirect effect of cautious sexual attitudes explains 99% of the impact of religious practice on intent to vaccinate against HPV (Figure 5).

### 3.4. Univariate Analysis

**Figure 6:**
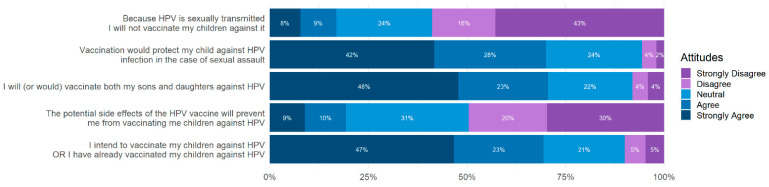
Outcome variables. Five survey items were used to measure attitudes toward the HPV vaccine and intent to vaccinate. The majority of respondents agreed with the statements “I intend to vaccinate my children against HPV OR I have already vaccinated my children against HPV”, “I will (or would) vaccinate both my sons and my daughters against HPV”, and “vaccination would protect my children against HPV infection in the case of sexual assault”. The majority of respondents disagreed with the statements “The potential side effects of the HPV vaccine will prevent me from vaccinating my children against HPV”, and “Because HPV is sexually transmitted I will not vaccinate my children against it”.

Five survey questions were used to determine an intent to vaccinate score. The proportions of the answers to these questions are shown in Figure 6. These answers were combined for each respondent and a total score used to indicate intent to vaccinate.

**Figure 7:**
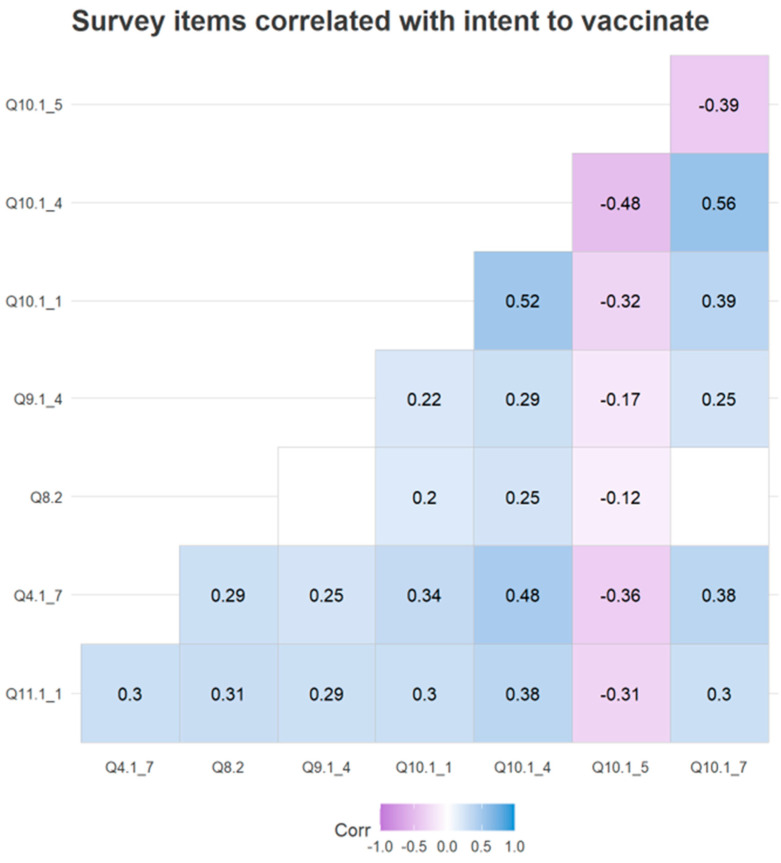
Survey items correlated with intent to vaccinate: A correlation matrix of the complete survey was created. Non-significant results and results with a low degree of correlation were removed to create an abbreviated correlation matrix. Blue coloration indicates a positive correlation with question 11.1.1 “I intend to vaccinate my children against HPV OR I have already vaccinated my children against HPV”, purple indicates a negative correlation, blank cells indicate that a result was not significant. Values are PCC correlation coefficients.

To determine which factors individually impacted intent to vaccinate a correlation matrix was created from the complete survey. Survey items were compared to question 11.1.1“I intend to vaccinate my children against HPV OR I have already vaccinated my children against HPV” which was used to represent parental intent to vaccinate their children against HPV. Questions 11.1.2, 11.1.3, 11.1.4, and 11.1.5 were excluded from analysis because they belong to the same latent variable as question 11.1.1 and are therefore highly correlated. Seven survey items were determined to be individually correlated with intent to vaccinate (Figure 7) (Table 4).

## 4. Discussion

### 4.1. General Attitudes toward Vaccines Are Predictive of Intent to Vaccinate

In both the combined structural model and partial structural model A, positive vaccine attitudes are the greatest predictor of intent to vaccinate (Figure 3 and Figure 4). Correlation matrix analysis supports this finding; survey items 10.1.1, 10.1.4, 10.1.5, and 10.1.7, which makeup the latent variable “positive vaccine attitudes”, were all correlated with intent to vaccinate (Figure 6). Both structural equation modeling and correlation matrix analysis show that positive vaccine attitudes increase parental intent to vaccinate against HPV, which is consistent with previous findings. In a previous study on Christian parents completed by our lab, we found that positive attitudes toward vaccines were the greatest predictor of parental intent to vaccinate their children against HPV [24]. Parents who already have positive attitudes about vaccines are likely to be comfortable giving their children all vaccines which are recommended by the CDC even if they do not feel that their children have a very high risk of contracting high-risk HPV. It also follows that parents who feel less positively about vaccines would have lower intent to vaccinate their children against HPV especially if they do not feel that HPV poses a very high risk to their children. If parents who feel positively about vaccines have concerns about the HPV vaccine they are more likely than their vaccine-hesitant peers to seek information from authoritative sources such as the CDC website or discuss their concerns with a pediatrician and/or primary care provider. In partial model A, trust in healthcare professionals positively impacts vaccine attitudes, which increases intent to vaccinate. The indirect effect of trust in healthcare professionals through positive vaccine attitudes explains 96% of the “trust in healthcare professionals” influence on intent to vaccinate, which means trust in healthcare professionals has an indirect effect on intent to vaccinate by influencing positive attitudes. Parents who trust in their primary care providers are probably more likely to follow their recommendations and get their children vaccinated against HPV. Because HPV vaccines are relatively new, parents may not be aware that they exist and be aware of the benefits they provide; If primary care providers have a good relationship with parents they will have an easier time educating and recommending HPV vaccination. Attitudes about vaccines in general have been influenced by the COVID-19 pandemic, with many sources of vaccine misinformation arising [25].

### 4.2. High Religious Practice Negatively Impacts Intent to Vaccinate by Influencing Sexual Attitudes

In structural model A (Figure 4), high religious practice was seen to negatively impact intent to vaccinate against HPV. Structural model B (Figure 5) explains this effect by showing that high religious practice positively influences cautious sexual attitudes which negatively impact intent to vaccinate. The indirect effect of high religious practice through cautious sexual attitudes explains 99% of the variance, this means that 99% of the negative impact of high religious practice on intent to vaccinate is due to the influence of high religious practice on cautious sexual attitudes. Parents with cautious sexual attitudes may not feel that it is necessary to vaccinate their children against HPV because the vaccine only protects against sexually transmitted strains, and they may feel like the risk of their children contracting sexually transmitted HPV is low. Alternatively, parents who have cautious attitudes toward sex may not do much research on sexually transmitted diseases and consequently not be aware of the harm HPV infection can cause. The Church of Jesus Christ of Latter-day Saints (LDS), which is the predominant religion in Utah, advocates for sexual abstinence before marriage and fidelity within marriage [26]. The LDS Church’s stance on sexual relations is likely to at least partially explain why parents in our study with high religious practice had more cautious sexual attitudes. A substantial portion of our population identified as LDS (40%) and are therefore likely to be cautious about sex and encourage their children to abstain from sex until marriage.

### 4.3. Knowledge about HPV Increases Intent to Vaccinate, and Sex Education Can Increase HPV Knowledge

High HPV knowledge was shown to be predictive of intent to vaccinate in the complete structural model and in partial structural model B (Figure 3 and Figure 5). This shows that in our sample parents who understand the risks of HPV infection intend to protect their children against HPV through vaccination. Educating parents and caregivers about HPV may be an effective mechanism for increasing intent to vaccinate and increase overall HPV vaccination rates. In our study we observed that a majority (72.8%) of respondents agree or strongly agree with the statement “Sexual education is a necessary part of school curriculum.” Intent to vaccinate is also positively correlated with belief that sexual education is a necessary part of school curriculum (Figure 7). Knowledge of HPV is a predictor of intent to vaccinate, and sexual education is a medium whereby information about HPV can be delivered. Sexual education curriculum usually focusses on reducing the risk of pregnancy and infection through education. If parents feel that sexual education is a necessary part of school curriculum, it follows they are aware of the risks that their children may encounter and strive to protect their children through various means including education and vaccination.

### 4.4. HPV Vaccination Is Viewed Positively in Our Sample Population

A majority (70%) of our sample agree or strongly agree with the statement “I intend to vaccinate my children against HPV or I have already vaccinated my children against HPV” (Figure 6). This is a promising outcome because it shows that there is high intent in our population to vaccinate against HPV. The HPV vaccine was originally only recommended for females, but the CDC later changed their recommendation to include everyone. Seventy-one percent of our sample indicated that they would vaccinate both their sons and their daughters. This shows that the initial recommendation that the HPV vaccine only be administered to females does not impact current intent of parents to vaccinate all their children. The eight percent of respondents who disagreed with the statement “I will (or would) vaccinate both my sons and daughters against HPV” likely do not intend to vaccinate any of their children against HPV. Half of our sample indicate that they would vaccinate their children despite potential side effects. This could indicate that they feel that HPV presents a far greater risk to their children than the potential risk posed by receiving the vaccine. Thirty-one percent of respondents indicated that they neither agree nor disagree with the statement, “The potential side effects of the HPV vaccine will prevent me from vaccinating my children against HPV.” Most parents do not know the frequency of adverse reactions to the HPV vaccine. Uncertainty about the frequency of adverse reactions could explain why a large portion of the sample is unsure if they agree or disagree with the statement about vaccinating their children despite potential side effects. Educating parents about the potential risks of vaccination and the frequency of adverse effects could help increase vaccination rates as they learn that adverse reactions are uncommon, and the risk of potential side effects is low.

### 4.5. Trust in Health Guidelines Increases Intent to Vaccinate

A majority (53.5%) of survey participants agree or strongly agree with the statement “I trust in public health guidelines provided by the CDC”. Trust in CDC public health guidelines is also positively correlated with intent to vaccinate (Figure 7). As of June, 2022, the Centers for Disease Control and Prevention (CDC) recommends that everyone be vaccinated against HPV at age 11–12. The CDC also recommends vaccination for everyone 26 and younger who were not adequately vaccinated when they were younger. Vaccination can be administered after 26 for high-risk individuals, or on the recommendation of a clinician [27]. Most HPV infections are mild and rarely cause life threatening complications therefore few parents are worried about their children contracting HPV. There is little discussion or public awareness about HPV and societal pressure supporting vaccination is low; therefore, institutions such as the CDC are often the only organizations promoting HPV vaccination. Because the CDC is the national agency responsible for health in the United States, trust in the CDC can reflect a broader trust in research and medical institutions. Trust in public health guidelines such as vaccine recommendations from the CDC can directly impact intent to vaccinate because the CDC and state and local health departments are often the only organizations encouraging people to get vaccinated against HPV

### 4.6. Belief That Vaccination Protects against HPV-Induced Cancer Increases Intent to Vaccinate

There is a positive correlation between intent to vaccinate and the belief that the HPV vaccine is effective at preventing HPV-induced cancer. This observation is consistent with the Health Belief Model, which is that if the perceived benefits are high (the prevention of cancer), the desire to perform the behavior (intent to vaccinate) is also high. Interestingly, only 15.1% of participants strongly agreed that the HPV vaccine prevents cancer, suggesting the lack of certainty about the causation and prevention of cancer. It is possible that if this sample was more educated about the ability of HPV to cause cancer, the intent to vaccinate would be higher.

### 4.7. Limitations

A limitation of this study is that it is conducted in a small state with a somewhat unique religious makeup. Therefore, although the results are interesting, there may be limitations to how generalizable they are.

## 5. Conclusions

In this study we were able to identify factors that influence the intent of Utah parents to vaccinate their children against HPV, and we were also able to determine the mechanisms by which these factors affect intent. This study shows that in Utah, positive attitudes about vaccines in general and knowledge about HPV have the greatest positive impact on the intent of parents to vaccinate children against HPV. We also found that high religious practice and cautious sexual attitudes have a negative impact on parental intent to vaccinate.

Parents who already feel positively about vaccination view the HPV vaccine as an effective method of protecting their children from HPV, have high trust in health care professionals, and had positive views of vaccination, in general. Parents who are knowledgeable about HPV understand the risks presented by infection which increases their intent to vaccinate and protect their children from infection. Parents with cautious sexual attitudes may feel that their children are unlikely to be exposed to the sexually transmitted strains of HPV that vaccines protect against, therefore they may feel that it is unnecessary to vaccinate their children against HPV. High religious practice has a negative effect on intent to vaccinate because of its considerable impact on cautious sexual attitudes.

Interventions focused on educating Utah residents about the risks HPV infection poses could improve HPV vaccine uptake. Interventions focused on addressing general concerns about vaccines would likely also improve HPV vaccine uptake.

## Figures and Tables

**Table 1:** Demographic Characteristics.

Range	Number	Percent of Total Responses
**Number of Children**	n = 365	
1	134	36.7%
2	116	31.8%
3	64	17.5%
4	33	9.0%
More than 4	18	4.9%
**Age**	n = 365	
18–25	23	6.3%
26–35	144	39.5%
36–45	155	42.5%
46–55	33	9.0%
Over 55	10	2.7%
**Gender**	n = 363	
Male	113	31.1%
Female	250	68.9%
**Ethnicity**	n = 365	
American Indian or Alaskan native	2	0.55%
Asian	10	2.74%
Black or African American	3	0.82%
Latino or Hispanic	30	8.22%
Pacific Islander or Native Hawaiian	8	2.19%
White	303	83.01%
Two or More	5	1.37%
Other/Unknown	2	0.55%
I prefer not to answer	2	0.55%
**Marital Status**	n = 364	
Single	46	12.64%
Married	249	68.41%
Partnered (non-married)	27	7.42%
Divorced	33	9.07%
Widow/Widower	6	1.65%
Other	3	0.82%
**Education**	n = 365	
Have not completed High School	9	2.47%
Finished High School	92	25.48%
Some College	100	27.40%
Associates degree	41	11.23%
Bachelors degree	84	23.01%
Post-baccalaureate/professional degree	38	10.41%
**Yearly Household Income**	n = 364	
Less than $25,000	46	12.64%
$25,000–$50,000	97	26.65%
$50,000–$100,000	157	43.13%
$100,000–$150,000	48	13.19%
$150,000–$200,000	15	4.12%
More than $200,000	1	0.27%
**Political affiliation**	n = 364	
Democrat	69	19.0%
Republican	137	37.6%
No political affiliation	122	33.5%
I prefer not to answer	27	7.4%
Other	9	2.5%
**Political leanings on social issues**	n = 365	
Very Liberal	25	6.8%
Liberal	29	7.9%
Somewhat Liberal	37	10.1%
Neither Liberal nor Conservative	134	36.7%
Somewhat Conservative	61	16.7%
Conservative	54	14.8%
Strongly Conservative	25	6.8%
**Religious Affiliation**	n = 364	
Buddhism	2	0.82%
Christianity	211	57.81%
Hinduism	3	0.82%
Islam	1	0.27%
Judaism	3	0.82%
Other	43	11.78%
No Religious Affiliation	101	27.67%

**Table 2:** Fit statistics for full measurement model.

Model (Latent Variables)	TLI	CFI	RMSEA	SRMR	χ2	Chi-Square Test df	*p*-Value
Combined model	0.886	0.900	0.057	0.076	887.029	408	<0.001

**Table 3:** Fit statistics for structural equation models.

Model (Latent Variables)	TLI	CFI	RMSEA	SRMR	χ2	Chi-Square Test df	*p*-Value
Combined model	0.885	0.902	0.050	0.067	1013.133	528	<0.001
Model A	0.916	0.929	0.070	0.062	398.635	143	<0.001
Model B	0.928	0.943	0.071	0.055	268.436	95	<0.001

**Table 4:** Survey items moderately or highly individually correlated with intent to vaccinate.

#	Text	PCC	*p*-Value
4.1.7	I trust in public health guidelines provided by the CDC (Centers for Disease Control and Prevention)	0.31	<0.0001
8.2	Sexual education is a necessary part of school curriculum	0.31	<0.0001
9.1.4	The HPV vaccine is effective at preventing almost all cancers caused by HPV	0.29	<0.0001
10.1.1	Vaccines are more helpful than harmful	0.30	<0.0001
10.1.4	Vaccines are extensively tested to ensure their safety	0.38	<0.0001
10.1.5	Vaccines contain dangerous toxins	−0.31	<0.0001
10.1.7	Vaccination efforts have considerably reduced the transmission of infectious diseases in the United States	0.30	<0.0001

## Data Availability

The complete dataset is available upon request and IRB approval.

## Supplementary Materials

The following are available online at https://www.mdpi.com/article/10.3390/vaccines10091382/s1, Table S1: Complete survey.
