# Supplementary material for: Cautious Sexual Attitudes Diminish Intent to Vaccinate Children against HPV in Utah
Source: Vaccines (Basel). 2022 Aug 24;10(9):1382. doi: 10.3390/vaccines10091382 (PMC9501815; doi:10.3390/vaccines10091382)
Supplement: Supplementary file 1 [file vaccines-10-01382-s001.zip › vaccines-1875257-supplementary.pdf]

Block 10

Title of the Research Study: Utah HPV vaccine attitudes
IRB ID#:IRB2022-165
My name is Brian Poole, PhD. I am a professor at Brigham Young University and I am conducting this research. You are being invited to participate in this research study about vaccination. I am interested to learn more about how you feel about the Human papillomavirus vaccine. Being in this study is optional. If you choose to be in the study, you will be asked to complete a survey, that should take approximately 15 minutes of your time. You can skip questions that you do not want to answer or stop the survey at any time. The survey is anonymous, and no one will be able to link your answers back to you. Please do not include your name or other information that could be used to identify you in the survey responses. You will receive your standard compensation from the survey provider for completing the survey.
Questions? Please contact Brian Poole at brian_poole@byu.edu or 801-442-8092. If you have questions or concerns about your rights as a research participant, you can call the BYU Human Research Protection Program at 801-422-1461 or BYU.HRPP@byu.edu. If you want to participate in this study, click the Accept button to start the survey.

Accept

Decline

Demographics

Do you currently live in Utah?

Yes

No

Do you have any children under the age of 15?

Yes

No

Number of children:

0

1

2

3

4

More than 4

What is your age?

Less than 18:

18-25

26-35

36-45

46-55

Over 55

Gender:

Male

Female

Non-binary/third gender

Prefer not to answer

Ethnicity:

American Indian or Alaskan native

Asian

Black or African American

Latino or Hispanic

Pacific Islander or Native Hawaiian

White

Two or more

Other/Unknown

I prefer not to answer

Martial Status:

Single

Married

Partnered (non-married)

Divorced

Widow/Widower

Other

Education:

Have not completed Highschool

Finished Highschool

Some College

Associate's Degree

Bachelor's Degree

Post-baccalaureate/professional degree (e.g. Masters, MD, DDS, PhD)

Yearly Household Income:

Less than $25,000

$25,000 - $50,000

$50,000 - $100,000

$100,000 - $150,000

$150,000 - $200,000

More than $200,000

What best describes your job or career?

Full time salaried

Full time hourly

Part time salaried

Part time hourly

Self employed contractor/ gig work (e.g. rideshare driver, online English teacher, multi-level marketing salesperson)

Student

Retired

Other (Please describe)

Political Affiliation:

Democrat

Republican

No political affiliation

I prefer not to answer

Other (Please describe)

Please indicate your political leanings on economic issues

Strongly liberal

Liberal

Somewhat liberal

Neither liberal nor conservative

Somewhat conservative

Conservative

Strongly Conservative

Please indicate your political leanings on social issues

Very liberal

Liberal

Somewhat liberal

Neither liberal nor conservative

Somewhat conservative

Conservative

Strongly Conservative

Religious Affiliation

Buddhism

Christianity

Hinduism

Islam

Judaism

Other

No Religious Affiliation

Specific Religious Affiliation (Buddhism)

Theravāda (Teaching of the Elders, Southern Buddhism)

Mahāyāna (Great vehicle, East Asian Buddhism)

Vajrayāna (Tantric/Esoteric Buddhism)

Navayāna

Other (please specify)

Specific Religious Affiliation (Christianity)

Anglican/Episcopalian

Baptist

Catholic

Christian (non-denominational)

Church of Christ/Disciples of Christ

Congregational

Jehovah's Witness

Church of Jesus Christ of Latter-Day Saints

Lutheran

Methodist/Wesleyan

Orthodox (Eastern)

Pentecostal/Charismatic

Protestant (Other)

Reformed/Presbyterian

Seventh-day Adventist

Other (please specify)

Specific Religious Orientation (Hinduism)

Vaishnavism

Shaivism

Shaktism

Smartism

Other (please specify)

Specific Religious Affiliation (Islam)

Shia

Sunni

Sufi

Other (please specify)

Specific Religious Affiliation (Judaism)

Conservative

Orthodox

Reformed

Other (please specify)

Specific Religious Affiliation (Other)

Jainism

Shintoism

Rastafarianism

Sikhism

Confucianism

Zoroastrianism

Pagan/neo-pagan

Traditional African

African Diaspora

Indigenous American

Aboriginal

Folk Religions (please specify)

Other (please specify)

Specific Religious Affiliation (No Religious Affiliation)

Agnostic

Atheist

Spiritual, but no specific affiliation

Other (please specify)

Select the county in Utah where you live:

I have lived outside of Utah for 3 or more years:

Yes

No

Where have you lived for the longest outside of Utah? (Please Specify)

Have you ever worked for a company that paid you exclusively based on your sales success and/or the success of those below you?

Yes

No

Have you ever been a representative, consultant, marketing affiliate, coach or business owner for a company that sells a product exclusively through representatives, consultants, marketing affiliates, coaches or business owners?

Yes

No

Which Language do you speak primarily at home?

English

Spanish

Tagalog

Mandarin

French

German

Navajo

Other (Please describe)

News Sources

I am well informed about national and international events:

Strongly agree

Somewhat agree

Neither agree nor disagree

Somewhat disagree

Strongly disagree

I am careful about the sources where I get my news:

Strongly agree

Somewhat agree

Neither agree nor disagree

Somewhat disagree

Strongly disagree

I check social media:

Multiple times a day

Once per day

A few times a week

Once per week

Less than once a week

Never

Which Social media site do you use the most?

Facebook

Instagram

Pintrest

Reddit

Snapchat

TikTok

Twitter

Youtube

I do not use social media

Other (Please describe)

The primary source where I get news is:

Local news station

Cable national news

Print news paper

Radio

News website or online journal

Social media

Which news station do you watch most frequently?

CNN

MSNBC

Fox News

Other (Please describe)

Which online news source do you check most frequently?

BBC

CBS

CNN

CNBC

ESPN

Forbes

Deseret News

Salt Lake Tribune

New York Times

Wall street Journal

Washington Post

Reuters

Other (Please Describe)

Trust in government

Please indicate how much you agree with the following statements about government.

	Strongly agree	Somewhat agree	Neither agree nor disagree	Somewhat disagree	Strongly disagree
I always vote in national elections	○	○	○	○	○
I always vote in local elections	○	○	○	○	○
I feel that elected officials actively work towards the good of the people.	○	○	○	○	○
I trust in the decisions of my local and state governments.	○	○	○	○	○
I trust in the decisions of the Federal government	○	○	○	○	○
I trust in public health guidelines provided by local and state health departments.	○	○	○	○	○
I trust in public health guidelines provided by the CDC (Centers for Disease Control and Prevention)	○	○	○	○	○
I feel that elected officials work to represent me regardless of whether they are the candidate I voted for or not.	○	○	○	○	○

Trust in Medicine

Please indicate how much you agree with the following statements about medicine:

	Strongly agree	Somewhat agree	Neither agree nor disagree	Somewhat disagree	Strongly disagree
Prescribed treatments are more beneficial than harmful	○	○	○	○	○
Doctors are competent, careful and well trained	○	○	○	○	○
Natural remedies such as essential oils are as effective at treating most conditions as prescription drugs.	○	○	○	○	○
Doctors often do not pay attention to, or disregard what their patients are telling them.	○	○	○	○	○
Natural remedies are often a better treatment for minor ailments than modern medicine.	○	○	○	○	○

Religious Practice

Please indicate how often you do the following activities associated with religious practice.

	More than once a day	Once a day	More than once a week	Once a week	More then once a month	Less than once a month	Never
How often do you read scriptures/holy texts?	○	○	○	○	○	○	○
How often do you attend Sunday School or religious classes/seminars?	○	○	○	○	○	○	○
How often do you pray?	○	○	○	○	○	○	○
How often do you attend organized worship services?	○	○	○	○	○	○	○
How often do you attend other activities sponsored by a religious group?	○	○	○	○	○	○	○

Expectations for children

The minimum education that I expect my children to obtain is:

Graduate from high school

Trade school

Complete some college

Complete an associates degree

Complete a bachelor's degree

Post graduate certificate or degree (e.g. Masters, PHD, MD DO, PA, DDS, JD ect.)

I do not have children

I have the same educational expectations for both my daughters and sons:

Strongly agree

Somewhat agree

Neither agree nor disagree

Somewhat disagree

Strongly disagree

I do not have children

How many hours a week is your child enrolled in extracurricular activities outside of school. (e.g. sports, music lessons, dance lessons, academic tutoring etc.)

10+ hours

5-10 hours

1-5 hours

less then 1 hour

My children are not currently enrolled in extracurricular activities

I do not have children

What type of school does your child attend OR what type of school do the majority of your children attend?

Public School

Charter School

Private School

Home School

I do not have children

Sexual attitudes

As a parent, I emphasize certain rules or cautions about sexual behavior: (If you are not a parent, indicate how you would emphasize this topic)

Excessively

Frequently

Something I teach but do no emphasize

Almost never

Never

Sexual education is a necessary part of school curriculum:

Strongly agree

Somewhat agree

Neither agree nor disagree

Somewhat disagree

Strongly disagree

I worry about outside sources (e.g. Social media, school, peers, entertainment) influencing my children's sexual attitudes: (If you do not have children please answer how you feel about children in general)

Strongly agree

Somewhat agree

Neither agree nor disagree

Somewhat disagree

Strongly disagree

To what extent are sexual relationships outside of marriage discouraged within your social group?

Strongly discouraged

Somewhat discouraged

Rarely discouraged

Not discouraged at all

As a parent, I plan to teach about sexual behavior outside of marriage or a committed relationship as follows: (If you do not have children, please answer how you would teach about this subject if you did)

Strongly Discouraged

Somewhat discouraged

Not discouraged

I do not plan to teach my children about sexual behavior

Other (please explain)

Sexually transmitted infections are very concerning to me:

Strongly agree

Somewhat agree

Neither agree nor disagree

Somewhat disagree

Strongly disagree

Knowledge about HPV

We would like to ask you a few questions about Human Papillomavirus (HPV) infection and disease. Please rate the following statements about HPV.

	Definitely true	Probably true	Neither true nor false	Probably false	Definitely false
HPV is the most common sexually transmitted infection in the United States	○	○	○	○	○
HPV infection can cause severe physical suffering	○	○	○	○	○
HPV causes cancer in women but not men	○	○	○	○	○
The HPV vaccine is effective at preventing almost all cancers caused by HPV	○	○	○	○	○
HPV infection is difficult to detect because most cases are mild or asymptomatic	○	○	○	○	○

Vaccine attitudes

Please rate how much you agree with the following statements about vaccines in general

	Strongly Agree	Somewhat agree	Neither agree nor disagree	Somewhat disagree	Strongly disagree
Vaccines are more helpful than harmful	○	○	○	○	○
Vaccines often have severe side effects	○	○	○	○	○
Vaccines are effective at preventing disease	○	○	○	○	○
Vaccines are extensively tested to ensure their safety	○	○	○	○	○
Vaccines contain dangerous toxins	○	○	○	○	○
My children are up to date on their recommended vaccines	○	○	○	○	○
Vaccination efforts have considerably reduced the transmission of infectious disease in the United States.	○	○	○	○	○

Outcome: Intent to vaccinate

Please indicate how much you agree with the following statements about the Human Papillomavirus (HPV) vaccine. HPV is a human virus that is primarily sexually transmitted. Vaccination for HPV is recommended for pre-teens before the expected onset of sexual activity.

	Strongly Agree	Somewhat agree	Neither agree nor disagree	Somewhat disagree	Strongly disagree
I intent to vaccinate my children against HPV OR I have already vaccinated my children against HPV	○	○	○	○	○
The potential side effects of the HPV vaccine will prevent me from vaccinating me children against HPV	○	○	○	○	○
I will (or would) vaccinate both my sons and daughters against HPV	○	○	○	○	○
Vaccination would protect my child against HPV infection in the case of sexual assault	○	○	○	○	○
Because HPV is sexually transmitted I will not vaccinate my children against it	○	○	○	○	○

I am vaccinated against Human Papillomavirus

Completely

Partially

I am not vaccinated against HPV

Powered by Qualtrics
